# Potential Transformation of Food Resveratrol: Mechanisms and Biological Impact

**DOI:** 10.3390/molecules30030536

**Published:** 2025-01-24

**Authors:** Ayoub Jaa, Patricia Homobono Brito de Moura, María Begoña Ruiz-Larrea, José Ignacio Ruiz Sanz, Tristan Richard

**Affiliations:** 1 University Bordeaux, Bordeaux INP, INRAE, Bordeaux Sciences Agro, OENO, UMR 1366, ISVV, F-33140 Villenave d’Ornon, France; ayoub.jaa@u-bordeaux.fr (A.J.); patricia.homobono-brito-de-moura@inrae.fr (P.H.B.d.M.); 2 Free Radicals and Oxidative Stress (FROS) Research Group of the Department of Physiology, Medicine and Nursing School, University of the Basque Country UPV/EHU, 48940 Leioa, Spain; mbego.ruizlarrea@ehu.eus (M.B.R.-L.); joseignacio.ruizs@ehu.eus (J.I.R.S.)

**Keywords:** *trans*-resveratrol, *cis*-resveratrol, δ-viniferin, transformation, biological properties

## Abstract

Resveratrol is a naturally occurring phenolic compound found in foods like grapes, berries, and peanuts. It has attracted substantial interest for its potential human health benefits, including antioxidant and anti-inflammatory effects. Research indicates that resveratrol may contribute to cardiovascular health, protect cognitive function, and exhibit anticancer properties. However, various factors such as pH levels, exposure to light, specific enzymes, and metal ions can alter its chemical structure, affecting its biological activities. These reactions can lead to the formation of different metabolites and polymers, which may affect the stability and bioactivity of resveratrol. This review examines the transformation of resveratrol from its natural sources to its consumption by humans. Additionally, we explore the biological activities of the resulting compounds of resveratrol transformations.

## 1. Introduction

*Trans*-resveratrol (3,5,4′-trihydroxy-*trans*-stilbene or resveratrol) is a natural compound from the stilbene family. Since its discovery in the roots of white hellebore (*Veratrum grandiflorum*) [1], it has been identified in a wide variety of plants [2,3]. In terms of dietary sources, it is most commonly associated with grapes and wine [4,5], where it has long been associated with the so-called “French paradox” [6]. However, *trans*-resveratrol can also be found in other foods, including berries, nuts, and chocolate [4,7]. This compound has generated significant interest due to its potential biological properties, including antioxidant and anti-inflammatory effects [8]. *Trans*-resveratrol exhibits, among other properties, neuroprotective, antidiabetic, anticancer, and anti-obesity activities [5,9].

Numerous synthetic methods can be employed to modify the molecular structure of resveratrol [10,11,12]. These reactions are primarily aimed at modulating its bioavailability and enhancing its biological effects. One strategy is to increase the number of hydroxyl groups forming a catechol or gallol group, improving both its water solubility and biological activity. Additionally, to further boost its bioactivity, various substituents can be grafted onto the molecule such as alkyl, prenyl, imine, amine, or halogen groups. Among the most commonly utilized reactions is methoxylation, which typically results in compounds that exhibit greater cytotoxicity than resveratrol itself. For instance, pterostilbene, a naturally occurring methoxylated derivative of resveratrol, displays cytotoxic activities that are often more potent than those of resveratrol [13]. Another modification involves the addition of glycosidic moieties through a glycosylation reaction. While the resulting compounds tend to be less biologically active, they generally have enhanced bioavailability [14]. Furthermore, upon ingestion, resveratrol undergoes various metabolic processes [15], leading to the formation of a variety of metabolites in both the gastrointestinal tract and other organs, including dihydroresveratrol, as well as conjugates with sulfate and glucuronate.

Nonetheless, before its consumption by humans, *trans*-resveratrol can undergo chemical transformations triggered by external factors such as pH or light exposure [16,17]. These reactions can compromise its stability, leading to new metabolites, including *cis*-resveratrol and δ-viniferin. Such transformations not only impact the stability of *trans*-resveratrol but may also affect its bioavailability and biological efficacy [18,19,20]. Despite the importance of these modifications, research on their occurrence in food matrices and the biological properties of the resulting compounds remains limited. This review examines the dietary sources of *trans*-resveratrol and the chemical transformations it may undergo before its consumption. Furthermore, we will highlight the potential biological properties of the primary compounds formed during resveratrol’s chemical transformation by external agents. Using the Scopus database, a bibliographic study was performed for the biological properties of resveratrol derivatives stilbenes formed by physicochemical processes (non-enzymatic ones). References were retrieved by searching the stilbenes by their names: *cis*-resveratrol, δ-viniferin, and 2,4,6-trihydroxyphenanthrene. After careful inspection, those articles not related to biological properties were discarded. The remaining references were included in the present review.

## 2. Food Sources

*Trans*-resveratrol (**1**) is a natural compound first isolated from the roots of white hellebore (*Veratrum grandiflorum*) [1]. In 1963, it was also identified in the roots of Japanese knotweed (*Polygunum Cospidatum*) [21], a plant widely recognized in the Chinese pharmacopoeia for its pharmacological properties [22]. *Trans*-resveratrol is the most well-known member of the stilbene family, which encompasses various phenolic compounds found in a wide range of plants [2,3]. This compound is present in several food sources, including berries such as blueberries, cranberries, and strawberries, as well as in nuts and chocolate [4,7]. However, grapes and wine are considered the primary dietary sources of resveratrol [4,5], with typical concentrations ranging from milligrams per kilogram of fresh weight.

## 3. Resveratrol Chemical Transformations

**Figure 1:**
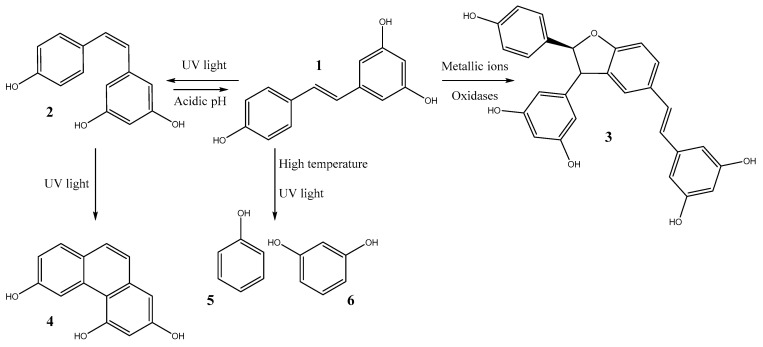
Chemical transformations of resveratrol highlighting *trans*-resveratrol (**1**), *cis*-resveratrol (**2**), δ-viniferin (**3**), 2,4,6-trihydroxyphenanthrene (**4**), phenol (**5**), and resorcinol (**6**).

Due to the inherent reactivity of the carbon-carbon double bond, *trans*-resveratrol (**1**) is susceptible to spontaneous chemical reactions, which can be initiated by environmental factors such as pH and light or catalyzed by agents like metal ions or peroxidases (Figure 1). These reactions can trigger degradation processes where the double bond breaks, resulting in the formation of monocyclic compounds like phenol (**5**) and resorcinol (**6**) [23]. Additionally, they may initiate isomerization reactions followed by cyclization, forming 2,4,6-trihydroxyphenanthrene (**4**) [17,24]. A notable example is the *trans*-to-*cis* isomerization of resveratrol under light exposure, leading to *cis*-resveratrol (**2**) [16]. Furthermore, oligomerization can occur in the presence of oxidative coupling agents [25,26] forming dimers such as δ-viniferin (**3**). In this section, we explore these various reactions in detail.

### 3.1. Effect of pH

The stability of *trans*-resveratrol is significantly influenced by pH. Under acidic conditions, this compound remains remarkably stable, even over extended periods. For example, at pH 1.2, no significant degradation is observed for over 90 days after solubilization in aqueous solution [27]. Within a pH range of 1 to 7, *trans*-resveratrol remains stable for at least 28 days [16]. However, as the environment becomes more alkaline, its stability decreases markedly. At pH 7.4 and 37 °C, its half-life is reduced to less than 3 days; at pH 8.0, the half-life decreases to under 10 h; at pH 10.0, it drops to less than 5 min [27]. This degradation is further accelerated at higher temperatures. While *trans*-resveratrol remains stable for over 90 days at −22 °C and a pH of 7.4, its half-life decreases to roughly 50 days at 4 °C and less than 3 days at 37 °C.

Interestingly, under alkaline conditions, the *cis* form of resveratrol proves to be more stable than its *trans* form [16,27]. At pH 10.0, the half-life of *cis*-resveratrol (**2**) is over 50 h. Conversely, at pH 1.0, *cis*-resveratrol (**2**) readily isomerizes back to *trans*-resveratrol, with more than 50% of the *cis* form converting to the *trans* form after 28 h.

At basic pH levels, the degradation of *trans*-resveratrol results in the formation of new yet unidentified compounds [14]. At high temperatures, however, resveratrol degradation leads to the formation of phenol and resorcinol [23].

### 3.2. Effect of Light

The *trans*-to-*cis* isomerization of resveratrol is well established [16,28], occurring when *trans*-resveratrol in solution is exposed to UV light, regardless of the solvent [29,30]. Upon UV radiation, *trans*-resveratrol converts to *cis*-resveratrol, with the conversion rate influenced by factors such as wavelength, light intensity, exposure duration, and the type of container used. For instance, in an alcoholic solution stored in a borosilicate glass container, over 90% of *trans*-resveratrol can be isomerized to its cis form within 80 min of exposure to 366 nm UV light, while only 63% conversion is observed after 10 h of exposure to 254 nm UV light [16]. This variation is attributed to the UV absorption properties of borosilicate glass. The resulting *cis*-resveratrol remains stable for at least 3 h under 366 nm irradiation [16].

Recent studies indicate that *cis*-resveratrol exposed to UVB radiation (wavelengths below 315 nm) can undergo further chemical transformations, yielding various secondary products [17,29,31]. One significant light-dependent reaction is photocyclization, which involves the intramolecular cyclization of the triene system via electronic excitation, resulting in the formation of the phenanthrene skeleton, characteristic of all stilbenes [31,32]. This process leads to the production of 2,4,6-trihydroxyphenanthrene (**4**) [17]. Additionally, this reaction can result in the formation of other compounds, including phenol (**5**) and resorcinol (**6**) [33].

Overall, the isomerization of *trans*-resveratrol occurs rapidly, with a half-life of under 10 min when exposed to sunlight, UVA, or UVB radiation [33]. The stability of *cis*-resveratrol is wavelength dependent [16,33]. While it remains stable in solution at 365 nm (UVA), its half-life is less than 1 h under solar exposure or UVB, although longer than that of *trans*-resveratrol [33]. Interestingly, recent studies propose utilizing the *trans*-to-*cis* isomerization of resveratrol as a potential method to protect the skin from UVA effects [30]. The kinetics formation of other compounds is also influenced by both the wavelength and duration of exposure [33].

The conversion of *trans*-resveratrol to *cis*-resveratrol has been observed in food matrices, particularly wine, for several years [28]. The concentrations of *cis*-resveratrol in wine are comparable to those of *trans*-resveratrol [5,34]. More recently, it has been demonstrated that *cis*-resveratrol can cyclize to form 2,4,6-trihydroxyphenanthrene in wine [24]. While *trans*-resveratrol, *cis*-resveratrol, and 2,4,6-trihydroxyphenanthrene remain stable in non-UV-exposed wine for over 100 days, exposure to UVB irradiation in a wine-like matrix results in rapid transformation. After 25 min, *cis*-resveratrol accounts for 20% of the initial *trans*-resveratrol concentration, while 2,4,6-trihydroxyphenanthrene reaches 76%. Notably, 2,4,6-trihydroxyphenanthrene concentrations of over 0.2 mg/L have been observed in UVB-irradiated wines after just 10 min of exposure in some wines [24].

### 3.3. Oligomerization

Under specific conditions, resveratrol can undergo oxidative coupling, leading to oligomerization reactions [35]. These reactions can be catalyzed by oxidases such as peroxidases [25] and laccases [36] as well as by metallic phenol oxidants like iron (III), silver (I), copper (I), thallium (III), or manganese (II) ions [25,37]. The nature of the products formed depends highly on the reaction conditions, encompassing factors such as the coupling agent, the solvent used, and temperature [25].

**Figure 2:**
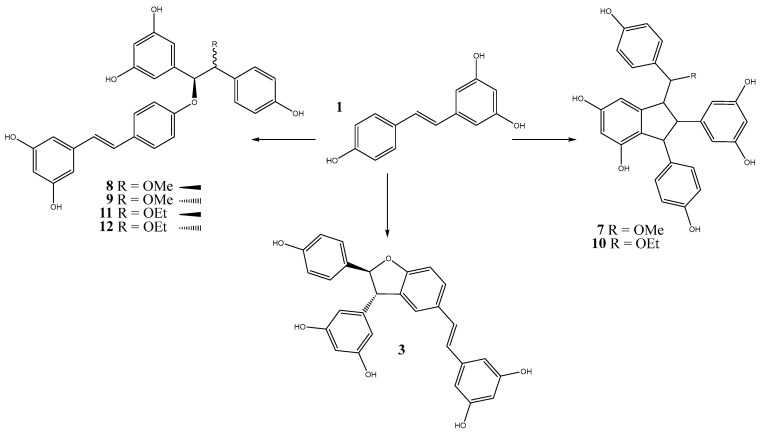
Compounds formed by the oligomerization of resveratrol in methanol and ethanol with silver acetate.

For instance, when resveratrol undergoes oxidative coupling in methanol with silver acetate as a catalyst (Figure 2), the predominant product is δ-viniferin (**3**), along with three additional compounds: parthenostilbenin B (**7**), oxystilbenin A (**8**), and oxystilbenin B (**9**) [38]. Similarly, in ethanol, the reaction yields δ-viniferin (**3**), as well as quadrangularin B (**10**), oxystilbenin F (**11**), and oxystilbenin G (**12**) [39]. Therefore, despite variations in the reaction medium, δ-viniferin consistently emerges as the primary product. For instance, in the presence of FeCl_3_ as a catalyst in acetone at 25 °C, the oxidative coupling leads to the formation of 97% δ-viniferin and less than 1% ε-viniferin after 20 h of treatment [25].

Notably, δ-viniferin is also naturally present in red wine [40]. Recent studies have demonstrated that this reaction can occur in food matrices like wine. For instance, heating wine at 30 °C for 24 h can lead to a fivefold increase in δ-viniferin concentration, accompanied by a corresponding decrease in resveratrol concentration [39]. This reaction is also associated with the formation of oxystilbenin G, a specific compound resulting from the oligomerization of resveratrol by oxidative coupling in hydroalcoholic solutions.

## 4. Biological Properties of Resveratrol Products

Numerous studies have explored the biological properties of *trans*-resveratrol, which has garnered extensive research interest due to its potential health benefits. In addition to its well-documented antioxidant and anti-inflammatory properties [8], *trans*-resveratrol exhibits neuroprotective, antidiabetic, anticancer, and anti-obesity effects [5,9]. Despite its low bioavailability and stability, *trans*-resveratrol is widely used as a dietary supplement, and current research efforts are focused on encapsulating it to enhance its stability and bioavailability [9]. In contrast, other stilbenes still need to be explored despite their therapeutic potential [41], likely because many of these compounds are not commercially available.

This review, rather than focusing on the well-known properties of *trans*-resveratrol, examines the potential biological effects of the primary compounds formed through its chemical transformations (Table 1).

### 4.1. Cis-Resveratrol

Among the compounds derived from the chemical transformation of trans-resveratrol, its cis isomer is the most extensively studied. However, the number of studies remains considerably lower than its trans form [65]. Interestingly, this compound is consistently found in concentrations comparable to trans-resveratrol in food products containing resveratrol [5,34].

Several studies have reported the anticancer and antiproliferative effects of *cis*-resveratrol across various cancer types (Table 1). For instance, Anoctamin 1 (ANO1), a calcium chloride-activated channel involved in cancer proliferation, migration, and invasion, was downregulated by *cis*-resveratrol in prostate cancer models [42]. Treatment with 30 µM of *cis*-resveratrol reduced ANO1 expression and inhibited its activity with an IC50 of 10.6 µM, resulting in a 97% reduction in PC-3 cell proliferation. Additionally, the compound inhibited cell migration dose-dependently and induced apoptosis through increased caspase-3 activity, PARP cleavage, and enhanced sub-G1 phase ratios. Interestingly, cis-resveratrol demonstrated a more potent inhibition of ANO1 activity compared to trans-resveratrol, as well as a stronger effect on inhibiting cell growth and migration in PC-3 prostate cancer cells [42].

Further evidence of anticancer and antiproliferative effects has been observed in studies targeting liver, intestinal, pancreatic, and renal carcinomas [43,44]. The *cis*-resveratrol in concentrations ranging from 10–25 μM showed an antioxidant effect on Caco-2 cells by modulating ROS production and inhibiting cell proliferation. Moreover, *cis*-resveratrol induced apoptosis by modulating the redox state and inhibiting the arachidonic acid cascade, eicosanoids production via the COX, 5-LOX, 12-LOX, and 15-LOX pathways, and the synthesis of hydroxyoctadecadienoic acids from the oxidation of linoleic acid [44]. *Cis*-resveratrol exhibited effects similar to those of trans-resveratrol, though they were generally less pronounced. However, these findings contrast with those reported by Hwangbo et al., who found that *cis*-resveratrol, isolated from Reynoutria japonica, inhibited DNA topoisomerase II activity but exhibited no cytotoxicity against human lung, ovarian, liver, and colon cancers, similar to the trans form [66]. Additionally, *cis*-resveratrol was ineffective against human medulloblastoma in contrast to the trans form [67,68].

The variability in *cis*-resveratrol’s anticancer effects may be attributed to cancer heterogeneity and different mechanisms of action [65]. Belleri et al. examined the biological activity of *trans*- and *cis*-resveratrol using various in vitro and in vivo models [69]. In contrast with *trans*-resveratrol, *cis*-resveratrol exhibited a limited inhibitory effect on the proliferation of bovine endothelial GM7373 cells. In chick embryo assays, it inhibited vessel formation by only 25% and exhibited minimal inhibition of CD31+ vessel numbers (14%) and CD31 mRNA levels in C57BL/6N mice.

This distinction in mechanisms of action has recently been emphasized. Due to the structural similarities with the tyrosine amino acid, resveratrol may act as a tyrosine antagonist by binding specifically to human tyrosyl-tRNA synthetase, an enzyme involved in protein synthesis [70]. Although both isomers bind to this enzyme, only *cis*-resveratrol appears to induce a structural modification that promotes interaction with poly-ADP-ribose polymerase 1 (PARP1), a key enzyme in cancer biology [65]. This interaction induces the activation of the protective stress response. On the other hand, *trans*-resveratrol inhibits the protective stress response mediated by tyrosyl-tRNA synthetase/PARP1 and induces the opposite effect compared with the *cis*-isomer [66]. These findings demonstrate that the two isomers may have different biological effects, underscoring the importance of investigating both isoforms [19].

In addition to its anticancer properties, *cis*-resveratrol exhibited various other biological activities. For instance, this compound has demonstrated cardioprotective properties by inhibiting platelet aggregation induced by collagen, ADP, and thrombin. However, its efficacy was lower compared to *trans*-resveratrol, as evidenced by higher IC_50_ values: 31 µM versus 15 µM for thrombin-induced aggregation, 16 µM versus 9 µM for collagen-induced aggregation, and 60 µM versus 25 µM ADP-induced aggregation [48].

Moreover, the metabolism of *cis*-resveratrol differs from that of *trans*-resveratrol [18]. Although both isomers produce the same metabolites, *trans*-resveratrol primarily results in human sulfate conjugates [26]. In a Transwell system using Caco-2 cell lines, *trans*-resveratrol preferentially formed sulfate conjugates, whereas *cis*-resveratrol produced glucuronide conjugates [18]. Additionally, the extent of metabolism for these two compounds varies significantly: 20% of *trans*-resveratrol is metabolized, compared to 62% of *cis*-resveratrol.

### 4.2. Other Compounds

Other resveratrol derivatives have received limited attention in research. However, recent studies have begun to highlight viniferins, the dimers of resveratrol, such as ε- and δ-viniferin [41,71]. δ-Viniferin, in particular, is recognized for its antifungal activities [72,73] and is one of the primary stilbenes synthesized in grapevine leaves in response to stress [74,75]. It is considered a major phytoalexin derived from grapevine resveratrol and is naturally present in wine [8]. Emerging research suggests that δ-viniferin may provide significant therapeutic benefits, including anticancer and antibacterial activities (Table 1).

One of the earliest studies examining the biological significance of δ-viniferin focused on the antitumor properties of a grapevine (*Vitis vinifera*) cell culture extract [56]. This extract demonstrated 8- to 10-fold greater antiproliferative activity on human breast cancer cell lines than trans-resveratrol alone by inducing a cell cycle arrest in phase S and thus, apoptosis in the HCC-1954 cell line [56]. Importantly, the extract showed no toxicity toward regular cell lines. The extract’s toxicity towards cancer cells was correlated with resveratrol dimers, including δ-viniferin.

The antitumor potential of δ-viniferin has been confirmed recently in various cell lines [52,53,55]. In lung cancer A549 cell line, δ-viniferin inhibited cell growth more effectively than *trans*-resveratrol [52], likely by reducing mitochondrial membrane potential (ΔΨm), increasing intracellular ROS level, and ultimately inducing apoptosis through the ROS/PI3K/Akt pathway.

In addition to its anticancer properties, δ-viniferin has demonstrated superior antibacterial activity compared to trans-resveratrol [61]. Interestingly, in this study, δ-viniferin was produced through oxidative coupling. Its antimicrobial activities have been observed against various pathogens, including *Escherichia coli*, *Bacillus cereus*, *Staphylococcus aureus*, and *Listeria monocytogenes* [61,62,63,76]. Finally, a recent study demonstrated that certain natural derivatives of δ-viniferine possess antiviral properties, effectively inhibiting several strains of influenza viruses and the SARS-CoV-2 Delta variant [77].

Other resveratrol-derived compounds resulting from chemical transformations remain underexplored, although dihydrophenanthrene 2,4,6-trihydroxyphenanthrene (**4**) has demonstrated antibacterial [64] and antioxidant activities [78]. The 2,4,6-trihydroxyphenanthrene induced a remarkable cytotoxicity on the *Caulobacter crescentus* and showed a genotoxic effect by increasing the *β*-galactosidase activity and increasing the oxidative DNA damage [64].

## 5. Conclusions

*Trans*-resveratrol is a bioactive compound present in a variety of foods, with well-documented biological properties. It can undergo chemical transformations influenced by external factors such as pH, light, specific enzymes, or metal ions, forming new compounds, such as *cis*-resveratrol and δ-viniferin. This review emphasizes that these transformations affect the stability of *trans*-resveratrol and may also alter its biological activities.

While the biological properties of *trans*-resveratrol have been extensively studied, its transformed derivatives, such as *cis*-resveratrol and δ-viniferin, have received less attention. However, emerging research suggests that some of these derivatives possess significant therapeutic potential, warranting further investigation.

From a chemical perspective, the mechanisms behind the formation of these compounds are well understood. Nonetheless, it would be valuable to investigate further and document their occurrence in various food products. For instance, while the *trans*-to-*cis* isomerization of resveratrol in wine is well-known and studied, the formation of compounds like 2,4,6-trihydroxyphenanthrene has been reported only once, indicating a gap in the literature.

Only preliminary studies, mainly in vitro, are available in terms of biological activity. Additional studies are necessary to better understand the mechanisms of action of resveratrol isomers. Currently, most research on δ-viniferin has been conducted in vitro. Given the relatively straightforward synthesis of δ-viniferin from *trans*-resveratrol, evaluating its bioactivity in vivo would be valuable, particularly for developing novel cancer therapies.

## Figures and Tables

**Table 1:** Biological activities of resveratrol products (↑: upregulation; ↓: downregulation).

Compounds	Biological Effects	Molecular Targets	Study Model	Dosis	Ref.
*cis*-resveratrol (**2**)	Anti-cancer	↓ cell proliferation and migration ↓ mRNA, ANO1 expression ↑ ROS, caspase-3, PARP cleavage, sub G1 phase and apoptosis	Prostate cancer (PC-3) in vitro	10–100 µM	[42]
		↓ cell proliferation	Hepatocellular/colon/pancreatic/renal carcinoma (HepG2, Hep3B, HCT-116), in vitro		[43]
		↓ COX-1, COX-2, 5-LOX, 12-LOX, 15-LOX, HODEs ↓ proliferation, ↑ATP release, ROS, apoptosis	Intestinal carcinoma cells (Caco-2)		[44]
	Anti-inflammatory	↓ ROS ↓ caspases-1 and -4	Human macrophages in vitro	1–100 µM	[45]
		↓ IL-1β, pro-IL-1β ↓ mRNA, COX-2, NOS-2	Rat macrophages in vitro		[46]
	Skin protection	Isomerization *trans*/*cis*	in vitro, in silico	–	[30]
	Antiplatelet	↓ platelet aggregation	Human plasma in vitro	1–10 µM	[47]
		↓ platelet aggregation thrombin, collagen and ADP	Rat plasma in vitro		[48]
	Protein-ligand interaction	↑ BSA	in vitro	0–20 µM	[49]
		↓ β-LG, α-LA, β-casein			[50]
	Antibacterial	*Escherichia coli* *Staphylococcus* sp.	in vitro	–	[51]
δ-viniferin (**3**)	Anti-cancer	↓ proliferation, ΔΨm, GR, GSH, PI3K/Akt/mTOR pathway ↑ ATP release, ROS, apoptosis	Lung Cancer A549 in vitro	0–100 µM	[52]
		↓ proliferation	Caco-2, HepG-2 cells		[53]
		↓ proliferation ↑ DNA damage ↑ epigenotoxic and cyto-genotoxic effects	A375, H460, PC3, WS1 cells	0–200 µM	[54]
		↓ proliferation Cell cycle arrest	Breast cancer MDA-MB-231 In vitro		[55]
		↑ S and G2/M arrest, apoptosis	*Vitis vinifera* extract HCC-1500, HCC-1954, MCF-7, HepG2 in vitro	0–125 µM	[56]
	Anti-inflammatory	↓ NO	Murine microglial BV2 cells	5–40 μM	[57,58]
	Cardiovascular	↓ Cytotoxicity and apoptosis ↓ ROS, Caspase-3, -7 and -9 ↑ MMP, SIRT1	Endothelial HUVECs cells	0.5–5 μM	[59]
	Neuroprotective	↓ Cytotoxicity ↓ NO	Murine macrophage RAW264.7, PC12 Cells	3–100 μM	[60]
	Antibacterial	*Bacillus cereus* *Staphylococcus aureus* *Listeria monocytogenes* Membrane disruption ↓ DNA gyrase activity	in vitro	1–200 μM	[61]
		*Listeria monocytogenes*		1–200 μg/mL	[62]
		*Staphylococcus aureus*, *Pseudomonas aeruginosa * *Escherichia coli*, *Proteus Hauser*, *Listeria monocytogenes* ↑ β-galactosidase activity, DNA damage		1–512 μg/mL	[63]
2,4,6-trihydroxy-phenanthrene (**4**)	Antibacterial	↑ β-galactosidase activity, DNA damage	*Caulobacter crescentus*	10 µM	[64]
